# Phenolics and related in vitro functional activities of different varieties of fresh waxy corn: a whole grain

**DOI:** 10.1186/s13065-021-00740-7

**Published:** 2021-03-01

**Authors:** Lirong Chen, Yuqiu Guo, Xiaoyue Li, Kuijie Gong, Kaichang Liu

**Affiliations:** Crop Research Institute, Shandong Academy of Agricultural Sciences, Jinan, 250100 China

**Keywords:** Fresh waxy corn, Polyphenol, Antioxidant activity, Hypoglycemic, Hypocholesterol

## Abstract

The polyphenol distribution in fresh waxy corns of different color varieties and their functional activities, which may be useful for treating various chronic diseases, were investigated. The in vitro antioxidant activity, and hypoglycemic and hypocholesterolemic effects of the free and bound corn phenolics were determined to evaluate the edible value of fresh waxy corn. The colored varieties contained more phenols than the common varieties (white and/or yellow). The total free phenolic acid content of the six varieties was 6637.73 µg/g DW (dry weight), which was slightly higher (*P* > 0.05) than that of the total bound form (6276.65 µg/g DW). The total free flavonoid content was 5850.09 µg/g DW, which was higher (*P* < 0.05) than that of the total bound form (4874.51µg/g DW). No bound anthocyanin was detected in the methanol extracts of the tested varieties. For all the varieties, free polyphenols contributed 86−100 % and 70−78 % of the 2, 2-diphenyl-1-picrylhydrazyl (DPPH) and hydroxyl radical scavenging abilities, respectively, and 100 % of the ferric reducing capacity. The free phenolics in fresh waxy corn showed better (*P* < 0.05) hypoglycemic effect than the bound form in terms of inhibition of *α*-amylase activity, whereas the bound phenolics of most varieties showed higher *α*-glucosidase inhibitory activity than the free forms. The free phenolics showed better (*P* < 0.05) glycocholesterol binding than the bound form for all varieties. The bound polyphenols showed better sodium cholate and taurocholate binding than the free form for most varieties. In conclusion, the difference between free and bound polyphenol content and functional activities indicates that fresh waxy corn can be potentially used for the development of functional food.

## Introduction

A growing body of evidence suggests that antioxidants or bioactive compounds are best acquired via whole-food consumption and not from dietary supplements [1]. Whole grains are now widely accepted as important factors mitigating the risk for type 2 diabetes, cardiovascular disease, and cancer [2–4]. Fresh waxy corn (*Zea mays* L. *sinensis* Kulesh), which is popular in East Asian countries, is also a whole grain. The tenderness of corn pericarp allows it to be consumed comfortably, thereby rendering corn intake an effective means of increasing dietary fiber, trace element, and vitamin content [5]. Therefore, immature corn grain can be used as a new raw material for the development of whole grain food and nutritional interventions suitable for human consumption [6]. Despite its differences with other whole grains, the strong natural acceptance of fresh waxy corn is attracting increased research attention [5, 7].

Accumulating evidence suggests that the benefits of phytochemicals, especially phenolics, in whole grains may be more than is currently understood as the oxidative stress induced by free radicals is involved in the etiology of a wide range of chronic diseases [1, 8]. Several studies have characterized polyphenolic compounds such as phenolic acids and flavonoids from corn [4, 6, 7]. Corn has the highest total phenolic content, followed by wheat, oats, and rice [4]. Correspondingly, the abundant phenolic content can promote the application of fresh waxy corn as a whole grain.

Phenolics in cereals are present in soluble-free, soluble-ester, and insoluble-bound forms [9]. When ingested, the free phenolics are rapidly absorbed by the small intestine, while bound phenolics survive gastrointestinal digestion but are released via colonic fermentation to exert their unique benefits in the colon [10–12]. In vitro digestion methods are useful for studying the release of these polyphenols and their stability under gastrointestinal conditions [7, 11]. Therefore, investigations on the distribution of both free and bound phenolic content and their related activities are key for understanding the potential health benefit of whole grain consumption [12]. Mature corn has been widely used to formulate products with improved functional activities owing to the presence of phenolics [13, 14]. However, information regarding the effect of fresh waxy corn on hyperglycemia, hyperlipidemia, and cancer is currently limited. Xu et al. [6] and Hu and Xu [7] have demonstrated the presence of free phenolics and related antioxidant activity in fresh waxy corn. However, detailed and comprehensive studies, especially regarding the differences between free and bound polyphenols, is currently lacking.

Therefore, the objectives of the present study were to investigate the polyphenol distribution in fresh waxy corn of different color varieties and study the in vitro functional activities of these phenolic compounds, which may be useful for treating various chronic diseases. The antioxidant activity, and hypoglycemic and hypocholesterolemic effects of the free and bound corn phenolics were determined to evaluate the edible value of fresh waxy corn. Our observations will facilitate production of nutraceuticals that will provide consumers increased access to the health benefits of fresh waxy corns.

## Materials and methods

### Material

**Fig. 1:**
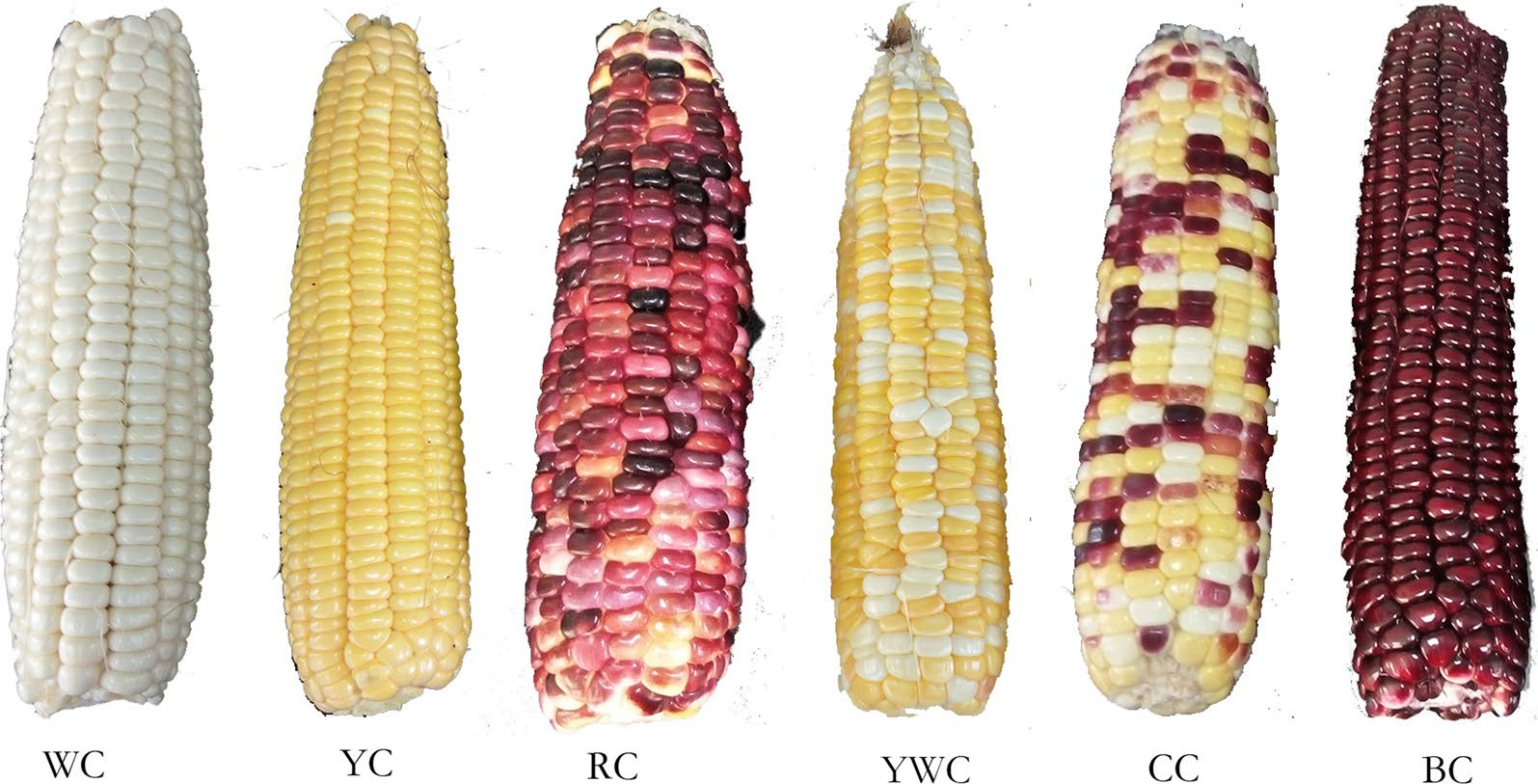
Tested varieties of fresh waxy corn

Different varieties of fresh waxy corn cultivars of various colors, namely, ‘Jingkenuo 2000’ (white corn, WC), ‘Xiameinuo’ (yellow corn, YC), ‘Caizhen 100’ (red corn, RC), ‘Minuo 4’ (yellow and white corn, YWC), ‘Huanuo 1’ (multicolor corn, MC), and ‘Yuheinuo 600’ (black corn, BC) were planted on an experimental farm at the Shandong Academy of Agricultural Sciences on June 8, 2018. Ears of corn that were 16−18 cm in length and 4.5−5 cm in circumference at their upper ends were harvested at 22−25d of milk stage post-pollination. The ears were then husked (Fig. 1) and five ears were used for various assays. The kernels from five ears were removed from the middle of the ear with a sharp knife. The kernels of each sample were mixed equally, frozen in liquid nitrogen, and stored at −80°C.

2,2-Diphenyl-1-picrylhydrazyl (DPPH), taurodeoxycholic acid, taurocholic acid, and glycodeoxycholic acid were purchased from Aladdin (Shanghai, China). *α*-Amylase and *α*-glucosidase were purchased from Sigma Chemical Co., Ltd. (St. Louis, MO, USA). All other reagents used were of analytical grade.

### Extraction of free and bound phenolics

Free and bound phenolics were extracted according to previous method [14]. The samples (5 g) were ground to flour and mixed with 100 mL chilled acidified methanol (95 % methanol and 1 M HCl 85:15, v/v). Extraction was performed at room temperature for 2 h in a water bath. The supernatants were obtained by centrifugation at 2500*g* for 10 min. The residue was re-extracted as mentioned above. The mixed supernatants were concentrated under vacuum at 45 °C and reconstituted to a final volume of 25mL.The product was stored at −20 °C and considered free phenolics.

The residue from the above free phenolic extraction was hydrolyzed with 200 mL 2 M NaOH at room temperature for 1 h with continuous shaking in the presence of nitrogen gas. The mixture was defatted with hexane and then neutralized with concentrated HCl. The remaining mixture was then extracted five times with ethyl acetate. The supernatants were combined and evaporated at 45 °C, and then reconstituted to a final volume 50 mL with chilled acidified methanol. The product was considered bound phenolics and stored at −20 °C until further analysis.

### Determination of phenolic acid content

Folin Ciocalteu (FC) colorimetric method was used to determine the phenolic acid content according to previous report [14]. The absorbance of the sample at 760 nm was measured and the phenolic acid content was expressed as gallic acid equivalent (GE) in the sample dry weight (DW).

### Determination of flavonoid content

The flavonoid content was determined and calculated by the absorbance of the sample at 510 nm according to previous report [14], and was expressed as (+)-catechin equivalent (CE) in the sample DW.

### Determination of anthocyanin content

The anthocyanin content was determined according to previous report [15] with some modifications. The absorbance of the methanol extracts was measured in triplicate at 535 nm in a 96-well microplate against an acidified methanol blank. Anthocyanin concentration was quantified by comparing the sample’s average absorbance to a calibration curve of cyanidin 3-O-glucoside chloride in acidified methanol. The final values were expressed in anthocyanin equivalents (AE) in µg/g DW.

### Determination of antioxidant activity

DPPH radical scavenging activity of phenolics was assessed by measuring the ability to bleach a black colored methanol solution of DPPH radicals as described by previous method [6, 16]. One milliliter phenolic extract was mixed with 5 mL 60 µM DPPH dissolved in methanol. The absorbance was measured at 517 nm against a solvent blank. The scavenging rate of DPPH radicals was expressed as the scavenging rate of one gram of dried samples and was calculated using Eq. (1).

1 $${\text{DPPH radical scavenging rate}}\left( {\% /g{\text{DW}}} \right) = \left( {\frac{{1 - {\text{Abs}}_{{{\text{sample}}}} - {\text{Abs}}_{{{\text{error correction}}}} }}{{{\text{Abs}}_{{{\text{control}}}} }}} \right) \times 100 \times \left( {\frac{{V_{{final}} }}{{V_{{test}} }}} \right) \div {\text{W}}_{{{\text{sample}}}}$$

where A_control_ is the absorbance of the control solution, A_sample_ is the absorbance in the presence of phenolic extracts in DPPH solution, and A_error correcting_, which is the absorbance of the extract solution without DPPH used for error correction. V_final_ represents the final volume of methanolic extract, V_test_ represents the volume used for activity test, and W_sample_ represents the total weight of sample DW.

Hydroxyl radical scavenging activity (HRSA) was assessed according to Mäkynen et al. [17] with some modifications. The reaction mixture was generated by adding 30 µL 2-deoxy-2-ribose (17 mM), 30 µL extract, 30 µL 1.2 mM EDTA, 60 µL 0.3 mM FeCl_3_, 30 µL 34 mM hydrogen peroxide (H_2_O_2_), and 60 µL 0.6 mM ascorbic acid. The reaction was performed at 37 °C for 1 h. Thereafter, 150 µL 1 % (w/v) thiobarbituric acid (TBA) and 300 µL 2.8 % (w/v) trichloroacetic acid (TCA) were added to the mixture, which was subsequently incubated at 100 °C for 15 min. The absorbance was measured at 532 nm against a blank containing deoxyribose and buffer. The HRSA values were expressed as scavenging rate per gram of dried samples and were calculated using Eq. (2).

2 $${\text{Hydroxyl radical scavenging rate}}\left( {\% /{\text{g DW}}} \right) = \left( {\frac{{{\text{Abs}}_{{{\text{control}}}} - {\text{Abs}}_{{{\text{sample}}}} }}{{{\text{Abs}}_{{{\text{control}}}} }}} \right) \times 100 \times \left( {\frac{{V_{{final}} }}{{V_{{test}} }}} \right) \div {\text{W}}_{{{\text{sample}}}}$$

where Abs_contol_ is the absorbance of the control solution (30 µL distilled water instead of extract) and Abs_sample_ is the absorbance in the presence of phenolic extracts. V_final_ is the final volume of the extract, V_test_ is the volume used for activity test, and W_sample_ is the total weight of sample DW.

Ferric reducing antioxidant power (FRAP) assay was performed according to previous report [16, 17]. Briefly, a FRAP solution was mixed with 10 mL 0.3 M sodium acetate buffer solution (pH3.6), 1mL 10 mM 2,4,6-tripyridyl-S-triazine (TPTZ) in 40 mM HCl, and 1mL 20 mM FeCl_3_. The FRAP reagent was warmed to 37 °C in a water bath. Next, 0.1 mL phenolic extract was mixed with 1.8 mL FRAP reagent and 3.1 mL ultrapure water. The absorption of the reaction mixture was measured at 593 nm after incubation for 30 min at room temperature. FRAP values were calculated from a standard curve prepared using FeSO_4_. FRAP values were expressed as µmol FeSO_4_ per gram of dried samples.

### **Inhibition assays for a-amylase and*α*-glucosidase activities**

The inhibition assays for *α*-amylase and *α*-glucosidase activities of extracts were according to previous report [14]. The absorance of the sample after reaction was measured at 540 nm by using a Shimadzu UV-2600 spectrometer. The results were expressed as inhibition (%) per gram of sample DW. The inhibition of *α-*amylase and *α*-glucosidase activities was calculated by the previous report [14].

### Determination of bile acid binding activity

The bile acid binding assay was performed according to previous method [17, 18] with some modifications. Sodium glycocholate, sodium cholate, and sodium taurocholate were used as bile acids in this experiment. Briefly, 1 mL of the extract was incubated with 1 mL 0.01 M hydrochloric acid solution with shaking at 37 °C for 30 min. The pH was adjusted to 6.24 and 4 mL of 10 mg/mL L-trypsin (prepared with 0.1 M phosphate buffer, pH 6.24) was added and incubated at 37 °C for 30 min at constant temperature. Four milliliters 1 mM cholate solutions (prepared with 0.1 M phosphate buffer, pH 6.24) was added to each sample. After shaking at 37 °C for 1 h, the mixture was transferred to a centrifuge tube and centrifuged at 3000×*g* for 20 min. The cholate content in the supernatant was analyzed. Bile acid binding activities were calculated from a standard curve using cholate.

### Statistical analysis

All the statistical analyses were performed using one-way analysis of variance (ANOVA) of the SAS 9.2 statistical software (SAS Institute Inc., USA). The data presented were the means of three experiments, along with the standard error of the mean. The means were compared using Fisher’s least significant difference (LSD) test, and differences at *P* < 0.05 were considered significant.

## Results and discussion

### Phenol contents of different varieties of fresh waxy corn

The polyphenol content of fresh waxy corn differed significantly (*P* < 0.05) among the varieties. The flavonoid content was relatively close to that of phenolic acids, whereas anthocyanin content was considerably lower than both (Table 1). The free phenolic acid and free anthocyanin contents ranged from 744.36 to 1907.70GAE µg/g DW and from 2.23 to 291.50 AE µg/g DW, respectively, which was in accordance with the ranges measured by Hu and Xu [7]. In terms of color, the colored varieties contained more phenols than the common varieties (white and/or yellow), except that the lowest flavonoid content of multicolor corn maybe due to the large number of white and yellow kernel (Fig. 1). The varieties with the highest content of phenolic acids, flavonoids, and anthocyanins were ‘Huanuo 1’, ‘Caizhen 100’, and ‘Yuheinuo 600’, respectively.

**Table 1:** Polyphenol content of different varieties of fresh waxy corn (µg/g DW)

Sample	WC	YC	RC	YWC	MC	BC
Phenolic acid						
Free form	1031.21 ± 33.70^bA^	744.36 ± 30.01^dA^	1165.73 ± 55.25^bA^	882.36 ± 17.91^cB^	906.37 ± 39.58^cB^	1907.70 ± 29.76^aA^
Bound form	1114.60 ± 59.56^bA^	415.65 ± 42.13^cB^	1190.60 ± 89.28^bA^	980.60 ± 21.30^bA^	2157.09 ± 65.97^aA^	418.11 ± 13.23^cB^
Subtotal	2145.81 ± 75.65^bc^	1160.01 ± 60.18^d^	2356.33 ± 145.99^b^	1862.96 ± 45.23^c^	3063.97 ± 116.39^a^	2325.81 ± 56.77^b^
Flavonoid						
Free form	872.89 ± 25.63^bcA^	1260.70 ± 47.58aA	1194.52 ± 51.20^aB^	685.47 ± 20.32^dA^	860.66 ± 29.55^cA^	975.85 ± 34.53^bA^
Bound form	833.11 ± 29.60^bA^	572.91 ± 6.59^cB^	1564.48 ± 89.22a^A^	624.99 ± 12.15^cA^	344.27 ± 7.10^dB^	934.76 ± 20.34^bB^
Subtotal	1706.95 ± 38.49^c^	1833.61 ± 47.97^bc^	2756.98 ± 153.24^a^	1310.46 ± 94.36^d^	1204.93 ± 38.52^d^	1910.61 ± 49.32^b^
Anthocyanin						
Free form	3.56 ± 0.53^d^	2.23 ± 0.31^d^	21.12 ± 0.88^b^	12.46 ± 0.89^c^	19.44 ± 1.57^b^	291.50 ± 8.233^a^
Bound form	nd	nd	nd	nd	nd	nd
Subtotal	3.56 ± 0.53^d^	2.23 ± 0.31^d^	21.12 ± 0.88^b^	12.46 ± 0.89^c^	19.44 ± 1.57^c^	291.50 ± 8.233^a^
Polyphenol form						
Free form	1907.55 ± 45.56^cA^	2006.12 ± 39.42^cA^	2382.23 ± 88.31^bB^	1578.93 ± 33.72^eA^	1788.32 ± 66.87^dB^	3174.11 ± 23.96^aA^
Bound form	1947.21 ± 77.30^cA^	988.39 ± 22.21^fB^	2754.79 ± 122.94^aA^	1605.52 ± 33.61^dA^	2498.73 ± 49.38^bA^	1351.84 ± 42.70^eB^
Total	3856.14 ± 111.72^c^	2994.56 ± 70.11^d^	5136.67 ± 168.75^a^	3184.49 ± 115.67^d^	4286.38 ± 125.32^b^	4524.44 ± 79.81^b^

All values are means of replicate determinations ± standard deviation (n = 3). nd indicates none detected. Different lowercase letters in the same row indicate statistical differences (*P* < 0.05) in the LSD test. Different uppercase letters in the same column of phenolic acid, flavonoid, or polyphenol indicate statistical differences (*P* < 0.05) in LSD test

The major portion of phenolics in grains existed in the bound form; for example, corn contains 85 % bound phenolics [4, 12]. However, in the present study, the total free phenolic content of the six varieties was 6637.73 µg/g DW, which was slightly higher (*P* > 0.05) than that of the total bound form (6276.65 µg/g DW). The total free flavonoid content was 5850.09 µg/g DW, which was higher (*P* < 0.05) than that of the total bound form (4874.51µg/g DW). No bound anthocyanin was detected in the methanol extracts of the tested varieties (Table 1). Therefore, we concluded that milk stage corn contained higher free phenols than the physiological mature corn. The abundance of free phenols in fresh waxy corn suggested that its functional activities were different from those of mature corn.

In terms of free phenol content, the order of the six varieties was YWC <MC<WC<YC<RC<BC. The free phenol content ranged from 1578.93 to 3174.11µg/g DW. In terms of bound phenol content, the order of the six varieties was: YC<BC<YWC<WC< MC< RC. The bound phenol content ranged from 988.39 to 2754.79 µg/g DW. The differences among varieties in the bound form content were larger than those of the free forms. ‘Yuheinuo 600’ and ‘Caizhen 100’ had the highest levels of the free and bound forms, respectively.

### In vitro **chemical antioxidant activity of polyphenol extracts of different varieties of fresh waxy corn**

**Fig. 2:**
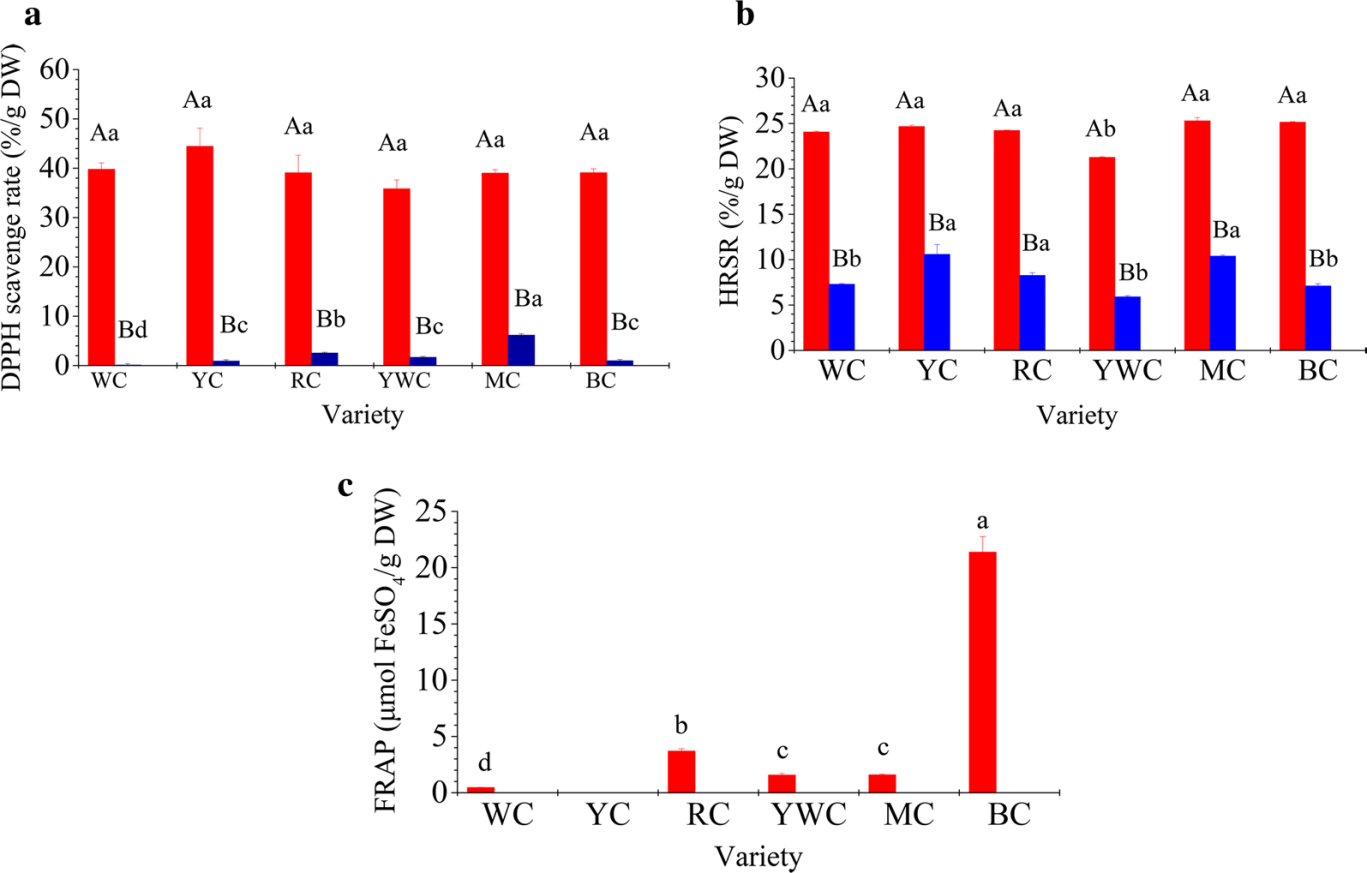
DPPH scavenge rate (**a**), hydroxyl radical scavenge rate (**b**), and FRAP (**c**) of the free ( ) and bound ( ) form polyphenols of different varieties of fresh waxy corn. All values are means of replicate determinations ± standard deviation (n = 3). Different uppercase letters in the same variety indicate statistical differences (*P* < 0.05) in the LSD test. Different lowercase letters in the different varieties of free or bound polyphenols indicate statistical differences (*P* < 0.05) in the LSD test

Corn possesses higher antioxidant activity than wheat, barley, rice, and oats [13]. In the present study, the differences in the antioxidant activity of different varieties and forms of polyphenols were evaluated using the DPPH and hydroxyl radical scavenging abilities, together with ferric reducing antioxidant power (FRAP). The free phenolics of different varieties showed higher (*P* < 0.05) antioxidant activity than the bound form (Fig. 2). No significant differences (*P* > 0.05) in DPPH scavenging rate was observed among the different varieties for the free form of polyphenols; the hydroxyl radical scavenging ability showed similar characteristics, except that YWC exhibited lower (*P* < 0.05) value than the other varieties. There was apparently no correlation between the radical scavenging activity and polyphenol content, which can possibly be because all radicals were scavenged by the different phenolic extracts despite differences in phenolic content among the varieties [6]. YWC contained the lowest free polyphenol content (Table 1), which suggested that it had the worst hydroxyl radical scavenging ability. The FRAP of free polyphenols differed from the radical scavenging ability, and significant differences among varieties were observed. The FRAP ranged from 0 to 21.23 µmol FeSO_4_/g DW, and BC showed the strongest ability than others, while YC showed the lowest ability without any ferric ion reducing power. Analysis revealed a significant (*P* < 0.05) positive correlation between FRAP and free anthocyanin content (R^2^ = 0.9876). This was because the experiment was conducted under acidic conditions, which stabilizes anthocyanins [15].

The bound form of polyphenols in different varieties showed significant differences (*P* < 0.05) in chemical antioxidant activity. Most varieties showed higher hydroxyl radical scavenging ability than DPPH scavenging ability for bound polyophenols, whereas the free forms showed completely opposite results. RC and MC contained the highest amount of polyphenol (Table 1), and correspondingly, both showed higher antioxidant activity (Fig. 2 aand b). Apart from this, significant correlation between phenolic contents and radical scavenging capacity was not observed.

In summary, free polyphenols contributed 86−100 % and 70−78% of the DPPH and hydroxyl radical scavenging abilities, respectively. The free polyphenols were responsible for 100% of the FRAP activity. This was considerably different from the results obtained with mature corn [4, 12], indicating that the difference between milk stage and mature period are due to differences in the polyphenol composition. Xu et al. [6] suggested that bound phenolics might contain highly reactive antioxidative substances. Das and Singh [12] considered free phenolics to be capable of participating in the FRAP reaction as much as bound phenolics owing to an electron transfer-based mechanism. However, the present results do not support these viewpoints.

Some studies have reported a correlation between polyphenol content and antioxidant activity [7, 12]. However, apart from the significant correlation between free anthocyanin content and FRAP, our results did not show a clear correlation between antioxidant activity and polyphenol content for any variety. Such inconsistencies have been reported previously [5, 19, 20]. Hu and Xu [7] indicated that these differences in correlation might be related to the types of materials, methods of assessing antioxidation, solvent extraction systems, and the complications associated with extracts containing two or more antioxidants.

### In vitro **hypoglycemic effect of phenol extract of different varieties of fresh waxy corn**

**Fig. 3:**
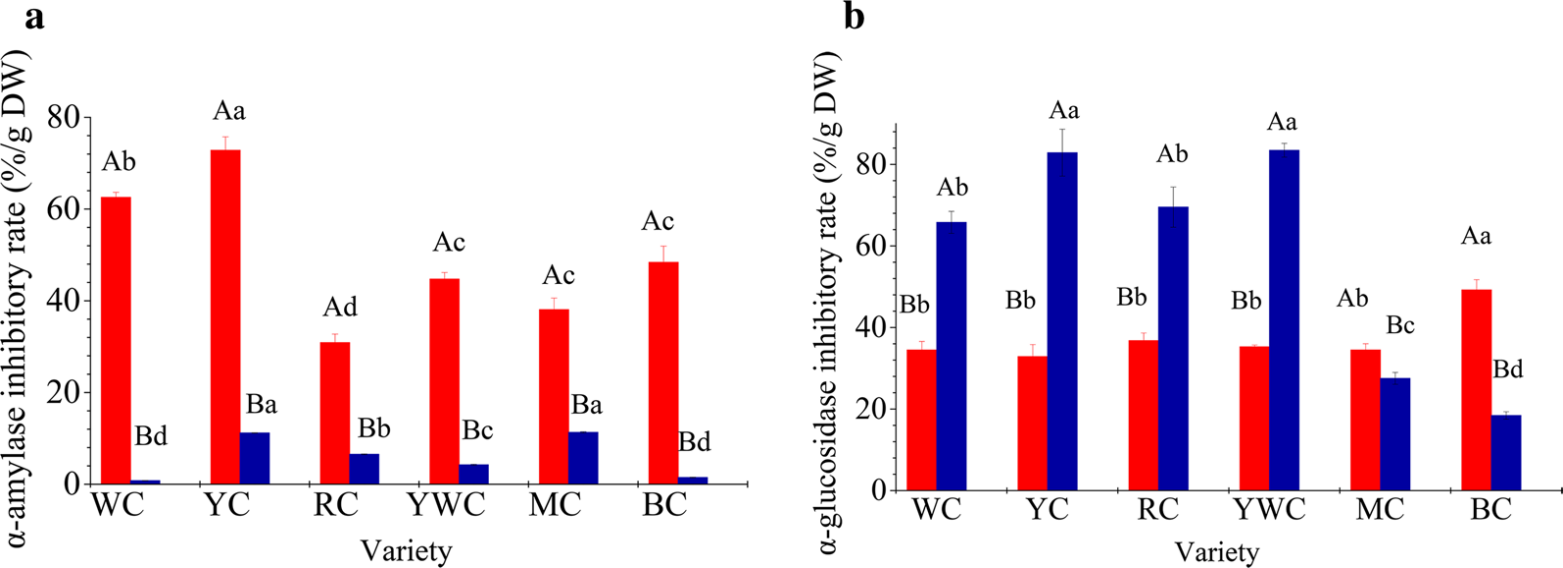
*α*-Amylase (**a**) and *α*-glucosidase (**b**) inhibitory activity of the free ( ) and bound ( ) forms of polyphenols of different varieties of fresh waxy corn. All values are means of replicate determinations ± standard deviation (n = 3). Different uppercase letters in the same variety indicate statistical differences (*P* < 0.05) in the LSD test. Different lowercase letters in the different varieties for free or bound polyphenols indicate statistical differences (*P* < 0.05) in the LSD test

Corn is considered as a potential value-added functional food ingredient that can reduce the risk for type 2 diabetes. Phenolic compounds have been considered another bioactive phytochemical that can control postprandial serum glucose levels and the incidence of type 2 diabetes [14, 21]. Phenolics, as inhibitors of pancreatic *α*-amylase and intestinal *α*-glucosidase, play important roles in inhibiting the rapid rise of postprandial blood glucose [2, 21, 22]. The free phenolics of fresh waxy corn showed better (*P* < 0.05) hypoglycemic effect than the bound form in terms of *α*-amylase activity inhibition, irrespective of variety (Fig. 3a). The inhibition rate of the free form was 5–60 fold higher than that of the bound form. Oboh et al. [23] reported similar results with jute leaf methanol extracts. On the contrary, bound form polyphenol of most varieties had higher inhibitory activity on α-glucosidase than the free form except MC and BC. The inhibitory rate of the other 4 varieties in the bound form was 1.9–2.5 times than that in the free form.

In terms of corn varieties, the free phenolic compounds of YC showed the highest *α*-amylase inhibitory activity, followed by WC, whereas RC showed the lowest *α*-amylase inhibitory activity. The other varieties exhibited similar activity. The bound phenolic compounds also showed significant differences among different varieties. YC showed strong *α*-amylase inhibitory activity, with a value of 72.82 ± 2.18 %/100 g DW for the free form and 11.18 ± 0.69 %/100 g DW for the bound form, whereas RC exhibited the lowest activity. The bound phenolic compounds also showed large differences in *α*-glucosidase inhibitory activity. YC and YWC possessed remarkable inhibitory activity, followed by WC and RC. In contrast, the *α*-glucosidase inhibitory activity of free phenolic compounds did not vary significantly among the varieties, with the exception of BC.

Oboh et al. [23] concluded that the *α*-glucosidase inhibitory activity of plant food was a function of their phenolic acid content, whereas Tadera et al. [24] indicated that flavonoids were stronger *α*-amylase inhibitors than other phenolic compounds. However, as shown in Table 1, the content of the free form of phenolic acids was not higher (*P* > 0.05) than that of the bound form for most varieties except BC. At the same time, the content of the bound form of flavonoids was not higher (*P* > 0.05) than that of the free form for most varieties except RC. Therefore, profile changes and some unidentified phenolic compounds might be responsible for our observations.

### In vitro **hypocholesterolemic effect of phenol extracts of different varieties of fresh waxy corn**

**Fig. 4:**
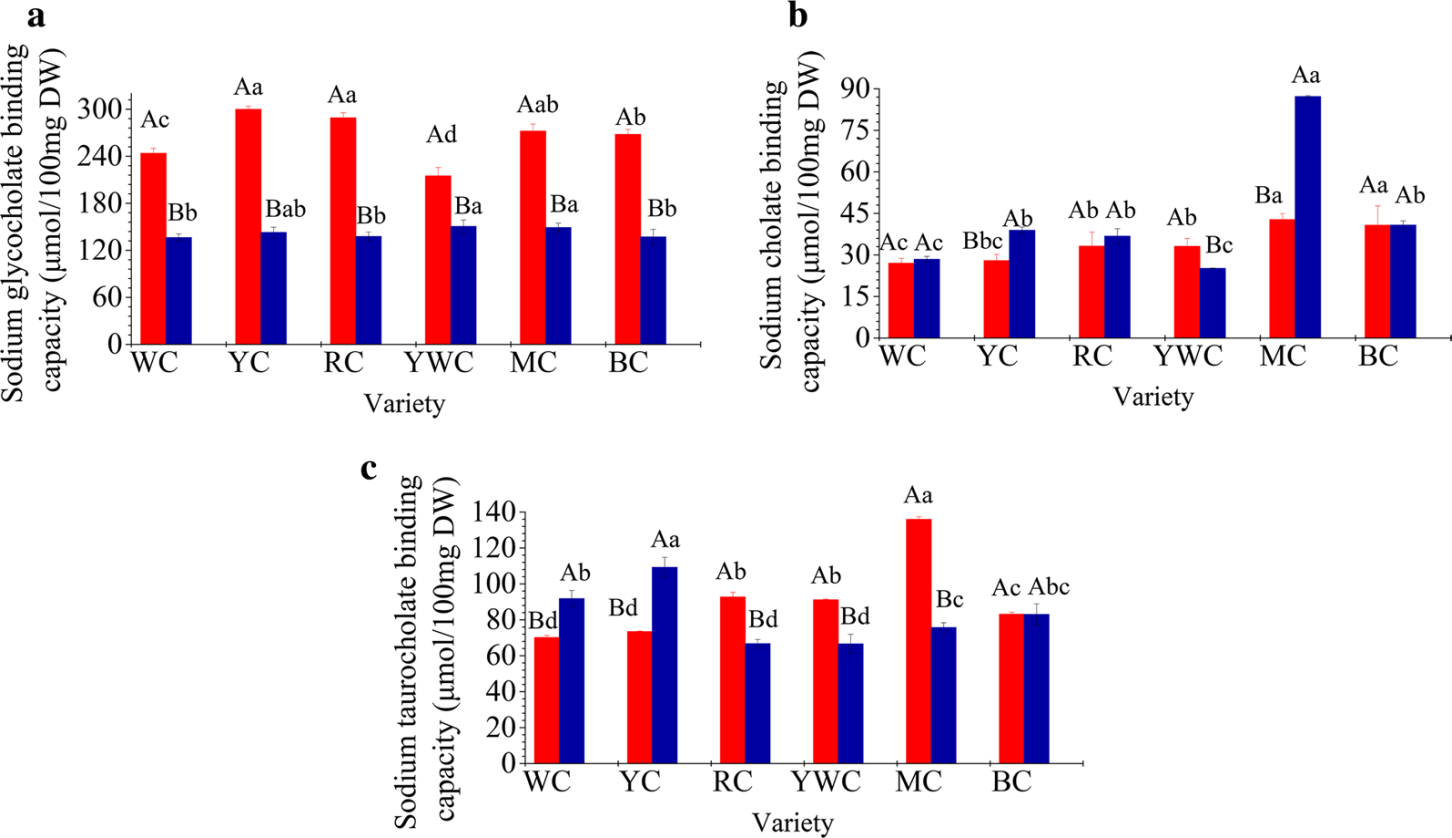
Bile acid binding capacity of the free ( ) and bound ( ) forms of polyphenols of different varieties of fresh waxy corn. All values are means of replicate determinations ± standard deviation (n = 3). Different uppercase letters in the same variety indicate statistical differences (*P* < 0.05) in the LSD test. Different lowercase letters in the different varieties indicate statistical differences (*P* < 0.05) in the LSD test. **a** sodium glycocholate binding capacity; **b** sodium cholate binding capacity; **c** sodium taurocholate binding capacity

Polyphenols can prevent reabsorption of bile acids in the small intestine by binding to them and triggering cholesterol destruction [25, 26]. Bansode et al. [27] concluded that low molecular weight polyphenols inhibited the intestinal transport of dietary cholesterol because of the decomposition of the bile acid-emulsified micellar structure in the intestines. In vitro bile acid binding activity is potentially related to the reduction in cholesterol content [18]. The methanol extracts of fresh waxy corn showed strong bile acid binding activity for all tested materials. The extracts showed the highest binding capacity for sodium glycocholate, with values above 130 µmol/100 mg DW (Fig. 4a), while the binding ability for sodium cholate was the lowest (Fig. 4b). The ability of the extract binding to sodium taurocholate ranged from 66.59 ± 5.36 to 135.93 ± 1.48 µmol/100 mg DW (Fig. 4c). Different forms of polyphenols showed considerable difference in bile acid binding capacity. The sodium glycocholate binding effect of free phenolics was 1.43–2.10 times than that of the bound form for all varieties (Fig. 4a), while the sodium cholate binding capacity of the bound polyphenols was 1.01–2.04 times than that of the free form, with the exception of YWC (Fig. 4b). The sodium taurocholate binding activity of the free forms in most varieties was 1.01–1.79 times than that of the bound form except WC and YC (Fig. 4c).

Previous studies have comprehensively determined bile acid binding capacity of some vegetables, fruits, cereal bran, and legumes [25]. Karataş and Sayar [26] observed that the bile acid binding value of fava bean seed coat was 37.50 ± 3.08 µmol/100 mg DW, which was lower than that obtained in the present study. However, most studies have used whole plants instead of extracts, and focused on dietary fiber instead of polyphenols. Studies on the bile acid binding ability of different forms of polyphenols are still limited.

Overall, MC showed the best bile acid binding activity for free and bound forms, whereas WC and YWC showed lower bile acid binding capacity. However, there was no significant correlation (*P* > 0.05) between the polyphenol content and the bile acid binding activity. Mäkynen et al. [17] obtained similar results in pomelo extracts. Condensed tannins [26, 28], insoluble dietary fiber [29], flavonoids [28], and lignin [30] or non-lignin components [31] were believed to be responsible for the bile acid binding activity. In fact, bile acid binding may be related to phytochemical, anionic, cationic, physical, and chemical structures, metabolite composition, and interaction with active binding sites [28, 32].

## Conclusions

The polyphenol content of fresh waxy corn differed significantly among the varieties. The free polyphenol content of most tested varieties was higher than or equally to that of the bound form with the exception of RC and MC. Free polyphenols possessed stronger antioxidant activity for all tested varities, contributing 70−100 % of the total antioxidant activity. The inhibitory effect of free polyphenols on *α*-amylase activity was stronger than that of the bound form, while the inhibitory ability of the two on *α*-glucosidase was opposite except two varieties. The free phenolics showed significantly better sodium glycocholate binding ability than that of bound form for all varieties, while the both showed equivalent sodiumtaurocholate and sodium cholate binding activity except one or two varieties. In summary, fresh waxy corn is an important whole grain product in health-promoting potential. To our best knowledge, this is the first comprehensive study of the polyphenol-related functional properties of fresh waxy corn in treating various chronic diseases. Further in-depth in vivo research is needed to better develop this emerging whole grain food resource.

## Data Availability

All data and materials are available on request (Lirong Chen and Kuijie Gong).

## Abbreviations

DW: Dry weight
DPPH: 2,2-diphenyl-1-picrylhydrazyl
WC: White corn
YC: Yellow corn
RC: Red corn
YWC: Yellow and white corn
MC: Multicolor corn
BC: Black corn
FC: Folin Ciocalteu
GAE: Gallic acid equivalents
CE: (+)-Catechin equivalents
AE: Anthocyanin equivalents
HRSA: Hydroxyl radical scavenging activity
TBA: Thiobarbituric acid
TCA: Trichloroacetic acid
FRAP: Ferric reducing antioxidant power
TPTZ: 2,4,6-tripyridyl-S-triazine
